# The Chromatin Landscape of Kaposi’s Sarcoma-Associated Herpesvirus

**DOI:** 10.3390/v5051346

**Published:** 2013-05-23

**Authors:** Zsolt Toth, Kevin Brulois, Jae U. Jung

**Affiliations:** Department of Molecular Microbiology and Immunology, Keck School of Medicine, University of Southern California, Harlyne J. Norris Cancer Research Tower, 1450 Biggy Street, Los Angeles, CA 90033, USA; E-Mails: brulois@usc.edu (K.B.); jaeujung@med.usc.edu (J.U.J.)

**Keywords:** Kaposi’s sarcoma-associated herpesvirus, KSHV, KS, viral chromatin, polycomb, EZH2, minichromosome, histone modifications, MLL/Set1, JMJD2A

## Abstract

Kaposi’s sarcoma-associated herpesvirus is an oncogenic γ-herpesvirus that causes latent infection in humans. In cells, the viral genome adopts a highly organized chromatin structure, which is controlled by a wide variety of cellular and viral chromatin regulatory factors. In the past few years, interrogation of the chromatinized KSHV genome by whole genome-analyzing tools revealed that the complex chromatin landscape spanning the viral genome in infected cells has important regulatory roles during the viral life cycle. This review summarizes the most recent findings regarding the role of histone modifications, histone modifying enzymes, DNA methylation, microRNAs, non-coding RNAs and the nuclear organization of the KSHV epigenome in the regulation of latent and lytic viral gene expression programs as well as their connection to KSHV-associated pathogenesis.

## 1. Introduction

It is estimated that 15% of human cancers are caused by viral infections. Among the seven currently known human oncogenic viruses, two of them belong to the herpesvirus family: Kaposi’s sarcoma‑associated herpesvirus (KSHV or Human Herpesvirus 8, HHV-8) and Epstein-Barr virus (EBV or Human Herpesvirus 4, HHV-4). In 1994, KSHV was first discovered in Kaposi’s sarcoma patients, who commonly present with cutaneous, neoplastic lesions of endothelial cell origin. Subsequently, it was also linked to the development of two B cell lymphomas: primary effusion lymphoma (PEL) and Multicentric Castleman’s disease [1,2]. Like other herpesviruses, KSHV establishes persistent infection in humans and alternates between two different life cycle phases: latency and lytic reactivation. In immunocompetent individuals KSHV establishes latency in CD19^+^ B cells, where a highly restricted viral gene expression program is thought to be the primary means by which latently infected cells escape detection by the host immune system. On the other hand, immune suppression along with other environmental and physiological factors, including oxidative stress, inflammatory cytokines, hypoxia or infection by other pathogens, each can favorably contribute to the physiological conditions required for KSHV to transition from latency to viral reactivation and virus production [3,4,5,6,7,8].

The 160-175-kb KSHV genome is composed of a single, 140.5-kb long unique coding region (LUR), which is flanked by 20-35-kb long, GC-rich terminal repeats (TRs) [9]. The LUR encodes at least 86 protein-coding genes, 12 microRNAs and several non-coding and antisense RNAs. The majority of latent genes are located between ORF69 and K14 in the KSHV genome and their expression is driven by only a few promoters. On the other hand, lytic genes are spread across the entire KSHV genome and the expression of each is likely regulated by over 100 different promoters. Latent genes are constitutively expressed in KSHV-infected cells, regardless of whether the infection is in a latent or lytic phase. On the other hand, lytic gene expression is repressed during latency and is only activated upon lytic reactivation, during which the three classes of lytic genes, immediate early (IE), early (E) and late (L) are induced, respectively [10]. While a relatively clear picture of the life cycle-dependent gene expression patterns of KSHV has emerged, we still do not understand the mechanism(s) that allow latent genes to escape the transcriptional repression affecting the rest of the viral genes during latency and the regulatory processes that control the genome-wide repression and temporally-ordered transcription of lytic genes. The fact that lytic reactivation of KSHV can be induced by treating latently infected cells with chemicals that affect chromatin regulatory factors such as histone deacetylases (HDACs), DNA methyltransferases (DNMTs) and histone acetyltransferases (HATs) argues that chromatin and chromatin-associated factors must be involved in the control of viral gene expression [11,12,13]. Indeed, whereas the KSHV genome is linear and histone-free in the viral capsid, after infection, the viral DNA becomes a closed circular episome that associates with cellular histones and persists in the nucleus as a non-integrated minichromosome [14,15]. Micrococcal nuclease mapping studies and chromatin immunoprecipitations of various histones and chromatin regulatory proteins have demonstrated that KSHV gene expression is controlled by the dynamic modulation of nucleosomal structure, a finding reminiscent of the regulation of cellular genes on the host chromosome (see below). 

**Figure 1:**
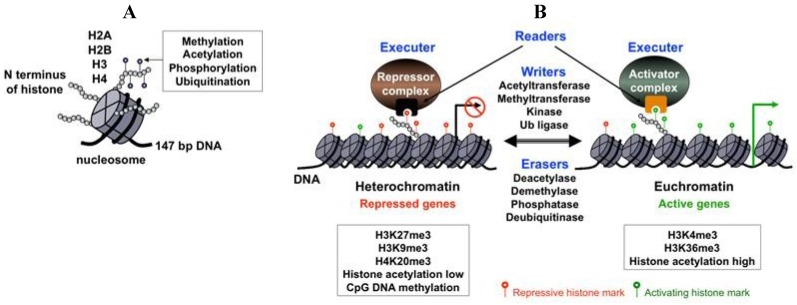
Chromatin components and their effect on gene expression. (**A**) The basic unit of chromatin is the nucleosome, which consists of 147 bp DNA wrapped around histones H2A, H2B, H3 and H4. The N terminus of histones is subject to different posttranslational modifications; (**B**) The levels and types of histone marks are dynamically regulated by antagonistic histone modifying enzymes (writers and erasers). Histone marks are recognized and interpreted by specific nuclear proteins (readers), resulting in the recruitment of either repressor or activator transcription regulatory complexes onto the target promoters. Some examples of repressive and activating chromatin modifications are listed in the boxed text.

The fundamental building block of chromatin is the nucleosome, which consists of 147 bp of DNA, which is wrapped around a histone octamer composed of two of each of the histones: H2A, H2B, H3 and H4 (Figure 1A). The N-terminal tail of histones protrudes from the nucleosomes and as such, is subject to various posttranslational modifications, including acetylation, methylation, phosphorylation and ubiquitination (Figure 1A). Depending on whether a gene is destined for activation or silencing, different histone modifying enzyme complexes are recruited to the gene promoter to generate specific posttranslational modifications on histones called histone marks, which function as key modulators of gene expression (Figure 1B) [16]. These histone marks can then be recognized by specific nuclear proteins that use histone mark recognizing modules [17]. These histone mark-readers can recruit additional transcription factors that either inhibit or activate transcription (Figure 1B). In addition to the histone marks, gene transcription can also be modulated by other structural features of chromatin, including the distribution of histone variants, the positioning of nucleosomes and the presence of cytosine 5-methylation of the CpG islands in the gene regulatory regions [18,19]. The nucleosome array of the DNA genome constitutes the primary structure of chromatin, which is further organized into a three-dimensional complex structure through expansive looping of the chromatinized DNA and large-scale compartmentalization in the nucleus [20]. This higher-order organization of the chromatin can influence genes within large chromosomal domains or co-regulate genes that are otherwise far from each other on the chromosome.

Chromatin immunoprecipitation combined with microarray technology (ChIP-on-chip), chromatin immunoprecipitation coupled with massively parallel DNA sequencing (ChIP-seq) and chromatin conformation capture assays have proved to be powerful techniques for the genome-wide mapping of histone marks, transcription factors and chromatin regulatory factors on the cellular genome and have provided a large amount data about the structural elements of the chromatin and their functional consequence for transcription. More and more studies have been emerging that also apply whole genome-analyzing tools for the interrogation of the epigenetic regulation of the KSHV genome, revealing an unexpectedly complex picture about the chromatin landscape of the viral genome in infected cells. In this review, we will mainly focus on the most recent studies that have investigated the regulation of chromatin on KSHV, including the relevant cellular and viral chromatin regulatory factors. Also, we will highlight some unanswered questions that require further investigation to better understand the fundamental chromatin-regulatory pathways involved in the control of the different stages of the KSHV lifecycle. 

## 2. *De Novo* Infection and the Requirements for the Establishment of Latency

**Figure 2:**
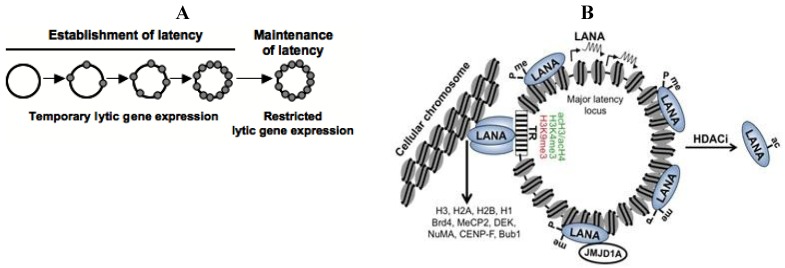
Chromatinization of the KSHV episome and the role of the latent KSHV protein, LANA. (**A**) The KSHV genome is linear and histone-free in the viral capsid and becomes a closed circular episome following *de novo* infection. Subsequently, the viral DNA is organized into a nucleosome structure and it persists in the nucleus as a non-integrated minichromosome; (**B**) LANA is a constitutively expressed gene that is encoded within the major latency-associated locus of the KSHV genome. LANA binds to terminal repeat (TR) region of the viral genome and tethers the viral genome to the host chromosome by interacting with histones or other components of the cellular chromatin such as Brd4, MeCP2…*etc*. LANA also binds to several sites within the viral genome and it is involved in the recruitment of the H3K9me1/2 histone demethylase JMJD1A and repression of lytic genes. The posttranslational modifications of LANA play a critical role in the association of LANA with the KSHV genome and also regulate its activity in transcription regulation. During latency, LANA in maintained in an arginine methylated state resulting in its binding to the KSHV genome. Upon HDAC inhibitor (HDACi) treatment, LANA gets acetylated, which leads to its dissociation from the KSHV genome and the concomitant induction of lytic genes.

KSHV can infect a variety of cell types, including endothelial, fibroblast and epithelial cells, and establishes predominantly latent infections in cultured cells. However, without continuous drug selection, KSHV episomes get lost from most proliferating cells and only a subpopulation of cells can stably maintain the viral genome. Grundhoff and Ganem showed that the establishment of latency in these cells is not due to genetic mutations in the host or viral genome, but presumably epigenetic changes on the viral episome [21]. Such epigenetic alterations could involve the nucleosome structure, DNA methylation or histone modifications depositing onto the KSHV genome in the nucleus. Although we are getting a more and more detailed picture of the chromatin architecture of the latent KSHV genome, we still do not understand the processes by which naïve, histone-free KSHV genomes get chromatinized following *de novo* infection (Figure 2A).

Establishment of latency requires repression of lytic genes and continuous expression of latent genes after *de novo* infection. This picture became more complicated when it was shown that a limited number of lytic genes with immunomodulatory and antiapoptotic functions are temporarily expressed after infection, followed by a decline in their transcription as latent gene expression remains constant (Figure 2A) [22]. Interestingly, during this transient period of lytic gene expression, one of the lytic genes that is expressed is ORF50, which encodes an IE protein called Replication and Transcription Activator (RTA). RTA is a viral transcription factor that functions as the master switch between latency and the lytic gene expression programs and is both necessary and sufficient for the initiation of lytic replication as it can activate several lytic promoters and the replication of viral DNA (reviewed in [23]). In addition, RTA binds to several chromatin and transcription regulatory factors, including the histone acetyltransferase CBP, the chromatin remodeling factor SWI/SNF2 and the Mediator, all of which are involved in gene activation [24]. Interestingly, despite the induction of RTA during *de novo* infection, its expression does not lead to full-blown lytic replication in this setting, possibly due to the lack of sustained RTA expression. While the mechanisms responsible for the down-regulation of RTA are unknown, they likely involve the rapid inhibition of the RTA promoter as well as the lack of the activation of specific signaling pathways. In agreement with this, infection of telomerase-immortalized retinal pigment epithelial cells with a recombinant KSHV clone that expresses RTA from the constitutively active cellular phosphoglycerate kinase promoter results in constitutive lytic replication [25]. Thus, it appears that the RTA promoter is inherently prone to become transcriptionally inactive following *de novo* infection, a process that likely involves certain RTA promoter elements that orchestrate the recruitment of transcription repressor complexes and heterochromatin-associated epigenetic marks such as DNA methylation and repressive histone modifications. Indeed, several repressive chromatin-associated factors have been identified on the RTA promoter during latency (see details below) but the molecular events involved in their initial recruitment upon *de novo* infection are still not known.

Since latent genes are rapidly induced after *de novo* infection and their expression persists during latency, it is plausible that latent proteins are involved in the repression of lytic genes. One of the latent proteins is the latency-associated nuclear antigen or LANA, which is a nuclear protein that binds to several sites on the KSHV genome, most notably the TR region (Figure 2B) [26,27,28]. It was shown that IE gene expression significantly increased in 293T cells following transfection of a LANA-deletion mutant of KSHV, suggesting that LANA plays a role in the downregulation of lytic genes during the establishment of latency [29]. Another group reported that HEK293 cells carrying a LANA-deletion KSHV mutant show increased expression of lytic genes, demonstrating that LANA can contribute to latency by repressing the expression of lytic genes [30]. The transcription repression activity of LANA can be attributed to its cellular binding partners, several of which are transcription repressors, including the heterochromatin protein HP1α, methyl-CpG-binding protein MeCP2, the H3K9me3 histone methyltransferase SUV39H1, histone deacetylase co-repressor mSin3 complex and DNA methyltransferases (DNMTs) [31,32,33,34,35,36]. LANA also interacts with and inhibits the enzymatic activity of the histone acetyltransferase CBP, resulting in repression of CBP target genes [37]. The functional consequences of the interaction of LANA with these chromatin regulatory proteins have been studied mainly in transcription reporter assays and not in the context of KSHV-infected cells. Therefore, it is currently unclear which of these protein-protein interactions has a role in the regulation of the viral chromatin and in repression of lytic KSHV genes.

Other important functions of LANA in the promotion of latency are the recruitment of cellular DNA replication factors onto TR and the tethering of the viral genome to the host chromosome during mitosis [14,38,39,40]. These LANA functions ensure that the replication of the KSHV genome occurs concurrently with that of the host genome and that viral episomes are disseminated to both daughter cells following mitosis. In accordance with this, a KSHV mutant lacking LANA was rapidly lost from cells after multiple cell divisions [41]. LANA interacts with histones H2A, H2B, H1 and cellular chromatin-associated factors such as Brd2, Brd4, MeCP2, DEK, nuclear mitotic apparatus protein (NuMA), centromeric protein F (CENP-F) and the kinetochore protein Bub1, all of which may be involved in the binding of LANA to the host chromosome [39,42,43,44,45,46,47]. Interestingly, LANA can associate with heterochromatin through binding to MeCP2 and this interaction might be involved in the downregulation of lytic genes during *de novo* infection, perhaps through tethering of the viral episome to a transcriptionally silenced host chromosomal regions [31,47]. Despite the ample number of cellular chromatin factors that bind to LANA, knowledge of the functional consequence of these interactions is limited, particularly in the setting of the LANA-mediated repression of lytic gene expression that occurs following *de novo* infection.

Posttranslational modifications of LANA also influence its function in the repression of lytic genes during latency by modulating its binding to the viral episome (Figure 2B). One group showed that arginine methylation in the histone-binding domain of LANA by the Protein arginine methyltransferase 1 (PRMT1) is associated with strong binding of LANA to the KSHV genome and repression of lytic genes [27]. Another group reported that treatment of KSHV-infected cells with histone deacetylase inhibitors causes the acetylation of LANA, which in turn results in the dissociation of LANA from the RTA promoter and the upregulation of RTA transcription [29]. Phosphorylation of LANA by Pim-1 and Pim-3 kinases can also counteract the transcription repressor activity of LANA on lytic genes [48]. Similarly, the phosphorylation of the histone-binding domain of LANA by different kinases can modulate association of LANA with the cellular chromatin [49]. These studies clearly show that the posttranslational modifications of the latent protein, LANA, play a critical role in the LANA-mediated inhibition of lytic genes during viral latency and presumably in the establishment of latency following *de novo* infection as well. 

## 3. Regulation of the Chromatin Structure of KSHV during Latency and Lytic Reactivation

During latency the KSHV genome exists as a circular episome in the nucleus and has a nucleosome structure similar to the bulk cellular chromatin (Figure 2A) [12,15]. Since chromatin restricts the accessibility of transcription factors to promoters, modification of the chromatin architecture has a pivotal role in the control of gene expression. Histone modifying enzymes and ATP-dependent chromatin remodeling complexes each play a role in chromatin remodeling through the covalent modification of histones and the repositioning of nucleosomes, respectively. There is evidence that both classes of chromatin regulatory proteins are involved in the regulation of KSHV gene expression. However, only a few studies have investigated the effect of these chromatin regulatory factors on the entire KSHV genome. Most of what we know about the chromatin structure of KSHV is based on studies performed with PEL cells that carry KSHV in latency and can be readily reactivated upon stress stimuli. Here, we summarize the most recent results regarding the role of histone modifications, histone modifying enzymes, DNA methylation, miRNAs, non-coding RNAs and the nuclear organization of the KSHV epigenome in the regulation of latent and lyitc viral gene expression programs.

### 3.1. Activating and Repressive Histone Modifications on the KSHV Genome

**Figure 3:**
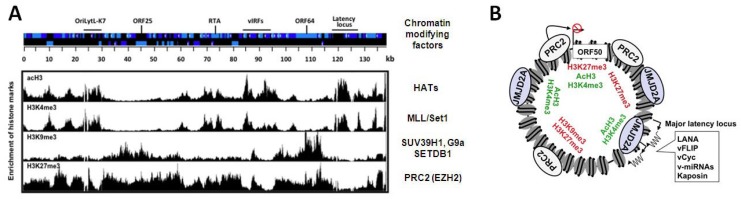
The chromatin landscape of KSHV during latency. (**A**) Schematic of the KSHV genome and the genome-wide distribution of different histone marks along the viral genome. There are distinct chromatin domains on the viral episome, which are characterized by different histone modification patterns indicating the targeted recruitment of specific cellular chromatin modifying enzyme complexes to different sites of the viral genome. The cellular chromatin modifying factors associated with each histone mark are listed to the right. The position of the latency locus and examples of an IE gene (RTA), some early genes (viral interferon regulatory factors or vIRFs and the OriLytL-K7 locus) and L genes (ORF25 and ORF64) are indicated at the top; (**B**) The PRC2 complex co-localizes with H3K27me3-rich chromatin domains, while the binding of the H3K9me3 histone demethylase JMJD2A overlaps with the H3K4me3/acH3-enriched chromatin regions that have a low level of H3K9me3. PRC2 is involved in the inhibition of ORF50 (RTA) expression during latency.

Since lytic genes are repressed during latency it was presumed that they are associated with heterochromatin that is poised for rapid and ordered transition to a transcriptionally permissive state. The histone modifying enzyme complexes involved in the regulation of specific genes can be determined by the identification of their corresponding histone modifications associated with the genes. Two studies have used chromatin immunoprecipitation assays in conjunction with high-resolution KSHV-specific tiling microarrays (ChIP-on-chip) to shed light on the nature of the chromatin that is associated with the KSHV episomes in infected cells [50,51]. Specifically, these techniques were used to analyze chromatin from a KSHV-infected B cell lymphoma cell line called BCBL1 and a latently infected adherent cell line called SLKp, providing the first genome-wide views of the KSHV epigenetic landscape, including the occupancy of total histone H3 and several of its modified forms: the activating marks, H3K4me3 and acetylated H3K9/K14 (acH3) as well as the repressive H3K9me3 and H3K27me3 histone modifications. While H3 turned out to be uniformly distributed, the histone modifications show distinct patterns along the KSHV genome (Figure 3A). Specifically, in certain regions of the KSHV genome, the genomic localization of H3K4me3 overlaps with acH3 and the majority of H3K27me3 co-localizes with H3K9me3 on the viral episome. In contrast, other regions of the genome showed mutually exclusive enrichment of activating and repressive histone marks. Latent genes have H3K4me3/acH3-rich chromatin during latency and reactivation, a finding that is consistent with the presence of transcriptionally active RNA polymerase II (RNAPII) on the latent promoters and their constitutively active transcription (Figure 3A) [50,52]. Interestingly, the chromatin of IE and the majority of E genes also displays high level of H3K4me3 and acH3 during latency, despite the absence of IE and E gene expression. During latency, the promoter region of the IE genes, RTA and ORF48, shows a characteristic bivalent chromatin, defined by the concomitant presence of H3K4me3 and H3K27me3 [50,51]. Although bivalent chromatin is not permissive for gene expression, it is associated with genes that are in a poised state of transcription activation, as is the case for IE and E genes. In contrast to the IE/E gene-rich genomic regions of KSHV, the parts of the viral genome that encode many of the late genes are mainly associated with the repressive H3K9me3 and H3K27me3 and these chromatin marks are presumably responsible for the inhibition of late gene expression during latency and also during the early phase of reactivation. High expression of late genes only occurs upon viral DNA replication and is accompanied by the dissociation of heterochromatin from the KSHV genome [50]. These observations imply that the activation of late gene expression may be triggered by the de-chromatinization of the KSHV genome upon viral DNA replication. In addition, recent studies with MHV68 showed that deletion of certain viral genes can abrogate the induction of late genes, without affecting viral DNA replication, suggesting that viral proteins with transcription regulatory activity are also involved in the regulation of late genes [53,54,55].

Strikingly, while H3K9me3 is restricted to two loci that encode mainly late genes, H3K27me3 is widespread across the entire KSHV genome, with the exception of the latency locus (Figure 3A) [50,51]. In addition, lytic reactivation leads to the decline of H3K27me3 on lytic genes, while the level of H3K9me3 remains constant [50]. In keeping with this, the H3K27me3 histone methyltransferase of Polycomb repressive complex 2 (PRC2), called EZH2, binds to the KSHV genome and colocalizes with H3K27me3 during latency. Upon reactivation, EZH2 dissociation from the KSHV genome is initiated and correlates with the concomitant decline of H3K27me3, increase of H3K4me3/acH3, and induction of IE and E gene expression [50]. A number of additional experiments were used to examine how viral gene expression is affected by the presence of EZH2 and its corresponding histone mark, H3K27me3 on the KSHV episome. The inhibition of EZH2 expression with either shRNA knockdown or the chemical compound, DZNep triggered the induction of lytic genes in latently infected PEL cells [50]. Conversely, the transient expression of enzymatically active H3K27me3 demethylases, such as UTX or JMJD3, could induce lytic genes in latently infected cells, while the H3K9me3 demethylase JMJD2A failed to do so [50,51]. These data suggest that the PRC2 complex maintains a H3K27me3-enriched heterochromatin on lytic genes to repress their expression during latency (Figure 3B). Moreover, viral reactivation triggers the dissociation of PRC2 from the KSHV genome and the concomitant deposition of activating histone modifications and RNAPII on viral promoters, resulting in the induction of the IE and E genes [50,51,52].

### 3.2. The Polycomb Connections

Polycomb group (PcG) proteins are cellular transcription repressors, which form two enzymatically distinct complexes called PRC2 and PRC1, both of which have been shown to inhibit transcription [56]. A number of different transcriptional silencing mechanisms contribute to PcG-mediated inhibition of transcription, including induction of chromatin condensation, inhibition of transcription initiation, blocking transcription elongation and recruitment of H3K4me3 histone demethylases to the target promoters [57,58,59,60]. PcG proteins are required for the inhibition of number of genes involved in development, cell proliferation and differentiation [56]. Importantly, there are several examples of human cancers in which increased expression of PcG proteins, specific point mutations or translocations of PcG gene loci have been observed [61]. Increased expression of specific PcG proteins have been shown to contribute transformation, indicating that PcG proteins can have oncogenic properties [62]. The PRC2 complex includes several subunits: EED, SUZ12, RbAp46/48, as well as EZH2, which catalyzes the di- and trimethylation of H3K27. In Drosophila, PRC2 has been shown to bind to target promoters through polycomb responsive DNA elements (PREs) [56]. In mammals, on the other hand, the recruitment of PRC2 to its target sites appears to be mediated mainly by various non-coding RNAs and distinct transcription factors [56]. The association of PRC2 with the chromatin is often extended beyond the target promoters in mammalian cells and overlaps with the distribution of H3K27me3 along the target genes. This was explained by the finding that PRC2 could be recruited to H3K27me3 via EED, a PRC2 subunit that can recognize and bind to this histone mark [63,64]. Similarly, the localization of the PRC2 components, EZH2 and SUZ12, on the KSHV episome is not restricted to specific sites but spread across the entire genome [50]. While we have a clear picture of PRC2 occupancy during the latent and early lytic phases of the KSHV lifecycle, the events that enable the initial recruitment of PRC2 to the KSHV genome following *de novo* infection and its maintenance on the latent genome are still not understood.

It has been shown that most polycomb target genes are repressed by both PRC2 and PRC1 complexes. Recently, a comprehensive proteomic and genomic analysis revealed that there are six major PRC1 complexes comprising a distinct set of proteins [65]. However, all six of these PRC1 complexes harbor a common subunit called RING1A/B, which are E3 mono-ubiquitin ligases that generate H2AK119ub. In most cases the binding of PRC1 to target genes depends on the activity of PRC2 and the presence of its corresponding histone mark, H3K27me3, a histone modification that is recognized by PRC1 and used for its recruitment. Nevertheless, it should be noted that PRC1 can also be recruited to number of target sites that lack H3K27me3 [66]. Indeed, the subunit composition of the individual PRC1 complexes affects their genomic localizations, indicating that different recruitment mechanisms exist for each PRC1 complex [65]. Specific components of both PRC2 and PRC1 have been found to interact with lytic promoters of Herpes simplex virus type 1 (HSV-1) during latency, suggesting that not only PRC2, but also PRC1 might be involved in the control the heterochromatin of KSHV [67,68]. However, whether PRC1 also binds to the KSHV genome and plays a role in the repression of lytic genes has not yet been addressed. 

Another interesting aspect of KSHV epigenetic regulation is the process by which PcG protein-mediated repression of lytic gene expression is reversed during reactivation. Importantly, the lysine 27 residue on histone H3 (H3K27) can either be acetylated (H3K27ac) or mono-, di-, or trimethylated. H3K27ac is associated with the activation of genes and is catalyzed by the histone acetyltransferases CBP/p300 in mammals [69]. The induction of lytic genes upon the overexpression of H3K27me3 demethylases UTX and JMJD3 in latently infected cells suggests that the modulation of posttranslational modifications of H3K27 must be critical for the regulation of lytic genes [50,51]. RTA, which binds to and activates number of lytic promoters during reactivation, has been shown to interact with CBP [24]. Furthermore, several RTA responsive promoters can be found in the viral genomic regions where H3K27me3 declines during reactivation, suggesting that RTA may recruit CBP to these sites, resulting in a transition from H3K27me3 to H3K27ac. In addition, the mixed-lineage leukemia (MLL) protein-containing MLL/Set1 complexes mediate H3K4me3 and, like RTA, increase at the same sites where H3K27me3 declines on the KSHV genome during lytic reactivation [50]. Moreover, MLL/Set1 complexes have been shown to interact with CBP as well as the H3K27me3 demethylase, UTX [70,71,72]. Therefore, it is possible that the H3K27me3 decline on IE and E genes during reactivation is the consequence of the recruitment of protein complexes to the viral promoters that can catalyze both demethylation and acetylation of H3K27 to induce lytic gene expression. In fact, a recent study showed that, a highly abundant KSHV non-coding RNA called polyadenylated nuclear (PAN) RNA can interact with the RTA promoter and recruit the H3K27me3 demethylases, JMJD3 and UTX, as well as the H3K4me3 histone methyltransferase, MLL2 [73]. Since PAN RNA expression is dependent on IE genes, especially RTA, these data suggest that PAN RNA-mediated recruitment of chromatin factors to the RTA promoter may be part of a positive feedback regulatory mechanism that perpetuates RTA expression during later stages of reactivation. However, given that the expression of RTA is induced prior to that of PAN RNA (RTA is an IE gene and PAN RNA is an E gene), PAN RNA is not likely to be involved in the initial de-repression of PRC2-mediated inhibition of the RTA promoter during physiologically relevant reactivation conditions, *i.e.*, in the absence of exogenous PAN RNA expression. Thus, the means by which repressive histone marks are initially removed from the RTA promoter is still not understood.

The treatment of latently infected cells with histone deacetylase (HDAC) inhibitors can also trigger the induction of RTA and leads to the dissociation of EZH2 and the decline of H3K27me3 on the RTA promoter [74]. Several HDACs have been implicated in the repression of PcG target genes and may play a role in deacetylation of H3K27ac, allowing its trimethylation [75,76]. In fact, EED was found to interact with HDAC1, 2 and 3 in a yeast two-hybrid screen, suggesting a possible connection between PRC2 and HDACs [76]. Importantly, the HDAC inhibitor, trichostatin A (TSA), can induce the activation of PcG-silenced genes, including the expression of KSHV lytic genes [12,76]. Thus, HDACs can also be involved in the maintenance of PRC2-mediated lytic gene repression by promoting H3K27 deacetylation, thereby allowing PRC2 to catalyze the trimethylation of H3K27. Further experiments are required to see whether there are any specific HDACs that have a role in the regulation of PRC2-maintained KSHV latency.

### 3.3. Regulation of the Heterochromatin Mark H3K9me3 on the KSHV Genome

H3K9me3 occupies two specific regions of the KSHV genome, both of which encode primarily late genes (Figure 3A) [50,51,77]. Although H3K9me3 does not seem to be a major repressive histone mark for lytic genes, several studies found that H3K9me3 and its associated chromatin regulatory factors have important regulatory roles during the KSHV life cycle. Several histone methyltransferases (HMTs) can catalyze the methylation of H3K9, including G9a, SUV39H1 and SETDB1 [78]. SUV39H1 and the H3K9me3-binding protein HP1 have been shown to interact with LANA, resulting in their recruitment to the TR and some lytic promoters of KSHV, where they are involved in heterochromatinization during latency [33]. Since LANA ChIP-seq and ChIP-on-chip experiments revealed a number of LANA-binding sites on the KSHV genome, it would be important to know whether SUV39H1 can also be recruited to these LANA-binding sites and thereby facilitate H3K9me3 [26,27]. In addition, other H3K9me3 HMTs can also be recruited to the KSHV episome by cellular factors. Indeed, Hsing-Jien Kung’s group has found that the cellular transcription repressor, KAP-1, is a novel regulator for KSHV latency, which is known to interact with SETDB1, a H3K9me3 HMT [79]. KAP-1 was found to be associated with a significant number of lytic promoters during latency, which becomes dissociated upon reactivation. Interestingly, KAP-1 is a substrate of the viral kinase encoded by ORF36 and the binding of KAP-1 to the KSHV genome is modulated by ORF36‑dependent phosphorylation [79]. The phosphorylation of KAP-1 causes the decline of its sumoylation, which decreases the ability of KAP-1 to bind to chromatin and repress genes. Importantly, the knockdown of KAP-1 by shRNA resulted in a 5-fold increase of RTA-mediated reactivation. Thus, KAP-1 could be one of the cellular transcription factors responsible for the recruitment of a H3K9me3 methyltransferase onto the KSHV genome, which is also involved in the inhibition of lytic genes during latency [79].

Two elegant studies have recently reported the genome-wide binding and functions of the H3K9 histone demethylases, JMJD2A and KDM3A/JMJD1A, on the latent KSHV genome, shedding light on the reasons behind the restricted localization pattern of H3K9me3 on the KSHV genome (Figure 2B, Figure 3B) [77,80]. One of the studies showed that shRNA knockdown of JMJD2A resulted in attenuated lytic reactivation, while overexpression of an enzymatically active form of JMJD2A facilitated the induction of lytic genes [77]. Strikingly, the binding sites of JMJD2A on the viral episome inversely correlate with the presence of H3K9me3 and overlap with the H3K4me3/acH3-enriched genomic regions [77]. These results indicate that binding of JMJD2A to the IE and E genes during latency may be involved in preventing H3K9me3-marked heterochromatin formation on their promoters, thereby facilitating H3K9 acetylation and priming of the IE and E genes for robust induction upon reactivation. Based on the survey of several viral proteins, an IE/E protein called K-bZIP (K8) was found to interact with JMJD2A [77]. *In vitro* demethylase assays revealed that K-bZIP can inhibit the demethylase activity of JMJD2A. Moreover, it was shown that K-bZIP can recognize the H3K9me3 moiety and thereby interfere with the binding of JMJD2A with H3K9me3. Furthermore, K-bZIP could also block the demethylation of H3K9me3 caused by overexpressed JMJD2A in 293T cells. Despite of the robust expression of K-bZIP H3K9me3 level does not change significantly on the KSHV chromatin during reactivation. Thus, the effect of K-bZIP on H3K9me3 must be limited to a subset of K-bZIP target genes in infected cells. In fact, K-bZIP expression in KSHV-negative cells causes a global repression of host genes and increased H3K9me3-marked heterochromatin formation on cellular genes, including host immune-related genes [77,81].

Izumiya’s group has recently reported that LANA forms a complex with KDM3A/JMJD1A, a cellular H3K9me1/2 histone demethylase, in KSHV infected cells [80]. The genome-wide occupancy of LANA and JMJD1A on the KSHV genome showed a high degree of overlap and was inversely correlated with the level of H3K9me2. Furthermore, depletion of LANA expression or overexpression of a JMJD1A-binding deficient LANA mutant in cells decreased the binding of JMJD1A to the viral episome, indicating that LANA recruits JMJD1A to the KSHV genome during latency. *In vitro* histone H3 peptide pull-down assays using purified LANA showed that LANA can interact with H3, H3K9me1 or H3K9me3 but not H3K9me2. Finally, the shRNA knockdown of JMJD1A resulted in decreased lytic reactivation, suggesting that JMJD1A is involved in the maintenance of H3K9 methylation-free chromatin on latent and a specific subset of lytic genes [80]. These studies altogether have shown that the regulation of H3K9me3 on the KSHV genome is indeed important for the proper induction of the lytic gene expression program [77,79,80].

### 3.4. H3K4me3 and the MLL/Set1 Family

Enrichment of H3K4me3 is typically present at the 5' end of transcriptionally induced genes. The first histone methyltransferase complex that can catalyze mono-, di- and trimethylation of H3K4 was identified in yeast and is called COMPASS (complex of proteins associated with Set1) [82,83]. The enzymatic subunit of COMPASS is Set1, which has at least 6 homologs in human, Set1A, Set1B, MLL1, MLL2, MLL3 and MLL4, all of which form different complexes and have distinct genomic localization [84]. In addition to their shared common subunits (Ash2, RbpBp5, Wdr5 and Dpy30), they differ in their enzymatic subunits and also have unique components. For instance, the tumor suppressor protein, Menin can only be found in complex with MLL1 or MLL2, while UTX, the Pax transactivation domain-interacting protein (PTIP), PTIP-associated protein 1 and nuclear receptor coactivator NCOA6 are exclusive components of the MLL3 and MLL4 complexes. In addition, Wdr82 is restricted to Set1A/Set1B complexes [84]. Whereas the Set1A/B complexes are the major H3K4 methylases responsible for the bulk H3K4me3 in mammalian cells, MLL1-4 play an important regulatory role for specific subsets of genes [85,86]. Mutations and random translocations of MLL genes are frequent occurrences in hematological malignancies like acute myeloid and lymphoid leukemia [87].

Regulation of H3K4me3 levels is important for controlling gene expression, particularly during lytic reactivation of KSHV, when lytic gene expression is rapidly induced. H3K4me3 is present on certain viral promoters during latency and is increased following reactivation (Figure 3) [50,51]. However, further studies are needed to identify the cellular factors that modulate H3K4me3 levels during the KSHV life cycle. It has been reported that while the binding of the core subunits of MLL/Set1 complexes, Ash2 and Wdr5, can increase on lytic promoters following reactivation, this was not observed on the latent LANA promoter [88]. Also, recruitment of Set1A could be detected on lytic promoters during reactivation but whether it is the sole H3K4me3 methylase responsible for the increase of H3K4me3 on lytic promoters has yet to be determined [88]. In addition, another group showed that MLL2 can interact with the RTA promoter through the viral non-coding PAN RNA, which raises the question whether distinct MLL/Set1 complexes can be targeted to different viral promoters [73]. Future studies using MLL/Set1 component-specific shRNAs as well as applying ChIP assays to test which of the unique subunits of MLL/Set1 complexes bind onto the KSHV episome will help to identify which of the MLL/Set1 complexes is responsible for the regulation of H3K4me3 on the KSHV genome during latency and following lytic reactivation.

There are a number of examples known of crosstalk between different histone modifications. For instance, H2B monoubiquitination at lysine 120 by the RAD6/BRE1 ubiquitin ligase complex is required for the methylation of H3K4 in human cells [89]. Furthermore, it was shown that the RNA polymerase II-associated factor (PAF) complex mediates the recruitment of RAD/BRE1 to the transcription machinery, where the monoubiquitination of H2B promotes Set1-mediated methylation of H3K4 in human cells [89]. Thus, it would be interesting to determine whether differential regulation of H2B ubiquitination is involved in the regulation of H3K4me3 levels on the KSHV episome. 

### 3.5. Histone Acetylation and Deacetylation on the KSHV Genome

Acetylation and deacetylation of lysine residues of histone tails have antagonistic effect on gene transcription. While acetylation of histones by histone acetyltransferases (HATs) unravels chromatin and activates transcription, histone deacetylases (HDACs) induce chromatin condensation and gene silencing [90,91]. Shortly after the discovery of KSHV, it was found that lytic genes of KSHV can be induced from latency by treating latently infected cells with various HDAC inhibitors, highlighting the importance of histone acetylation in the regulation of lytic gene expression. Histone acetylation can occur at more than 20 different lysine residues positioned within histones. To date, 19 different HATs are known and each one is part of distinct transcription activator complexes. The majority of HATs can acetylate histones at multiple positions and likewise, the same lysine residue can be acetylated by various HATs. This redundancy makes it difficult to pinpoint the HAT(s) involved in a given histone acetylation on the KSHV chromatin (Figure 3A). Two ChIP-on-chip studies revealed that H3K9/K14ac are enriched on several lytic promoters during latency and are further increased upon lytic reactivation [50,51]. Using luciferase reporter assays, several cellular transcription factors that interact with HATs or HDACs (e.g., C/EBPα, AP1, Oct-1, STAT3 and RBP-Jκ) have been implicated in the regulation of specific lytic promoters such as RTA [23,92,93]. Nevertheless, comprehensive studies of histone acetylation on the KSHV epigenome during latency and reactivation are needed in order to provide more detailed insight into the role of histone acetylation and ultimately pave the way toward the identification of the HATs involved in viral gene regulation during the KSHV life cycle.

**Figure 4:**
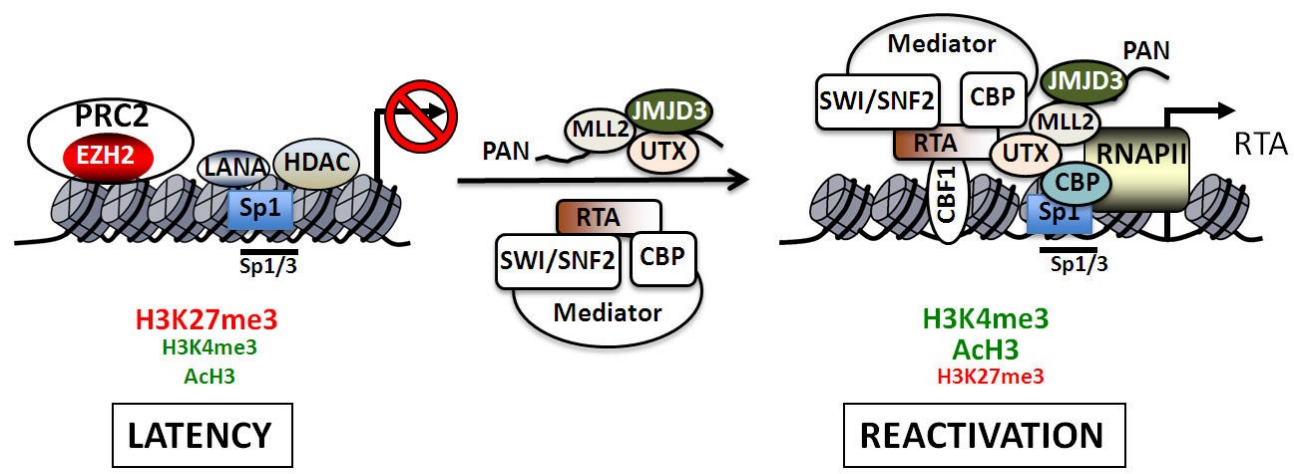
Regulation of the RTA promoter during latency and reactivation. The H3K27me3 histone methyltransferase complex PRC2 as well as specific histone deacetylases (HDAC1, 5 and 7) are critical transcriptional repressors that are present on the RTA promoter during latency. The bivalent chromatin of the RTA promoter is indicated by the presence of both activating (H3K4me3 and acH3) and repressive (H3K27me3) histone marks. After reactivation RTA binds to its own promoter via CBF-1 (also called RBP-Jκ) and recruits several cellular transcription factors such as the Mediator, the ATP-dependent chromatin remodeling complex SWI/SNF2 and the histone acetyltransferase CBP. The induction of transcription is accompanied by the rapid recruitment of RNA polymerase II (RNAPII) and increased binding of Sp1 to the promoter. The KSHV non-coding PAN RNA can also interact with the RTA promoter and recruit the H3K4me3 histone methyltransferase MLL2 as well as the H3K27me3 histone demthylases UTX and JMJD3. The Sp1/3-binding sites play an important role in the HDAC inhibitor-mediated reactivation of the RTA promoter. While these sites contribute to the LANA-mediated repression of the RTA promoter during latency, they are also involved in recruitment of CBP, which can catalyze the hyperacetylation of histones on the viral promoter during reactivation.

The interplay between HATs and HDACs in the regulation of RTA transcription was examined in detail. Lieberman’s group analyzed the chromatin structure of the core promoter of RTA in the presence or absence of the HDAC inhibitors (HDACi) such as sodium butyrate (NaB) and trichostatin A (TSA) [12]. They found that the RTA promoter is highly inducible by these HDACs and a NaB responsive element was mapped to a short GC-box DNA sequence that binds Sp1 and Sp3. Micrococcal nuclease (MNase) mapping and restriction endonuclease accessibility assays revealed that there is a stably positioned nucleosome at the transcriptional initiation site of RTA during latency, which rapidly changes upon NaB treatment [12]. Furthermore, it was also observed that HDAC1, 5 and 7 bound to the RTA promoter, resulting in hypoacetylation of the viral chromatin during latency, while reactivation resulted in the hyperacetylation of histones H3 and H4 concomitantly with the recruitment of components of the SWI/SNF2 ATP-dependent chromatin-remodeling complex onto the RTA promoter (Figure 4). Ectopic expression of the CREB-binding HAT, CBP, resulted in 10-fold induction of the RTA promoter as determined by luciferase reporter assay, whereas expression of other HATs such as p300, PCAF, GCN5 and TIP60 had no such effect. The CBP-mediated promoter stimulation depends on the Sp1/3-binding site, suggesting that CBP can be recruited to the RTA promoter via interaction with Sp1/3 during reactivation, resulting in the acetylation of both H3 and H4 [12]. In agreement with this, others also showed that NaB treatment could induce an Sp1-binding site-dependent recruitment of CBP and p300 onto the RTA promoter. Moreover, the increased Sp1-binding on the viral promoter is transient during reactivation [94]. The role of CBP and SWI/SNF2 in the regulation of the RTA promoter was further detailed by Gwack and his co-workers [24]. They showed that the C-terminal activation domain of RTA forms a complex with the Mediator, SWI/SNF2 and CBP in cells. During reactivation RTA recruits these cellular factors onto the RTA promoter and other RTA-responsive lytic promoters resulting in their transcriptional activation [24]. These studies altogether show that reactivation of lytic genes requires the recruitment of HATs by cellular and viral transcription factors to the viral promoters, resulting in the remodeling of the chromatin structure of the virus (Figure 4). 

Histone acetylation and nucleosome positioning also play a critical role in the regulation of the chromatin structure of the origin of latent replication (Ori-P), which is embedded in the GC-rich TR region of the KSHV genome. Ori-P contains two LANA-binding sites, where LANA binds and recruits the cellular DNA replication proteins, ORC2 and MCM, to facilitate the replication of the viral genome concomitantly with the replication of the host genome during mitosis [38,40]. It was shown that Ori-P is enriched with hyperacetylated histones H3 and H4 and that this enrichment is due to the activity of LANA, which recruits HATs such as HBO1 or CBP, to the Ori-P (Figure 2B) [40]. In addition, the bromodomain-containing cellular protein, Brd2, which binds to acetylated histones and is a known LANA-binding protein, is also recruited to the Ori-P, and may also be involved in the maintenance of the hyperacetylation of histones in Ori-P. Nucleosome mapping assays revealed a highly ordered nucleosome array in Ori-P that becomes disorganized in a cell cycle-dependent manner [40]. The current model is that the maintenance of a hyperacetylated chromatin in Ori-P facilitates the assembly of the DNA replication complex.

### 3.6. DNA Methylation of the KSHV Genome

In mammalian cells, DNA methylation occurs mainly on cytidine residues in the context of CpG dinucleotides. Methylated CpGs are often found in clusters called CpG islands [95]. Hypermethylated CpG islands in the 5' regulatory regions of genes are associated with gene silencing. Methylation of DNA inhibits gene expression either by impeding the binding of transcription factors to the promoter or by the action of methyl-CpG-binding proteins (e.g., MeCP2), which bind to methylated CpG islands and recruit HDACs and other transcription repressors to the gene to turn off its expression [96]. The maintenance of DNA methylation during cell division is mediated by DNA methyltransferase 1 (DNMT1), which copies the DNA methylation pattern to the daughter strands during DNA replication, while DNMT3a and DNMT3b are *de novo* DNA methyltransferases [97].

It has been shown that the treatment of PEL cells with the DNA methyltransferase inhibitor 5-AzaC can induce lytic reactivation of KSHV, suggesting that DNA methylation of lytic promoters is involved in the suppression of lytic genes during latency [11]. Recently, the global DNA methylation pattern of KSHV was determined in different latently infected cell lines by using immunoprecipitation of methylated DNA (MeDIP) in conjunction with KSHV specific high-resolution tiling microarray [51]. These experiments showed that KSHV is subject to extensive DNA methylation during latency. Like the H3K27me3 pattern on the latent KSHV genome, DNA methylation was excluded from the transcriptionally active latency-associated locus, while most of the lytic genes were associated with DNA hypermethylation. Surprisingly, the RTA promoter was not methylated in most of the cell lines, suggesting that DNA methylation is unlikely to be involved in the inhibition of RTA expression during latency [51]. In addition, while the latency-specific histone modification patterns were rapidly deposited on the viral episome following *de novo* infection, DNA methylation patterns were established comparatively slower, indicating that DNA methylation does not play a role in establishment of latency. On the other hand, DNA methylation may reinforce the inhibition of lytic genes at late timepoints of infection [51]. 

### 3.7. Nuclear Organization of the KSHV Genome

It is known that the KSHV episome is tethered to the cellular chromosome by LANA, which interacts with histones and several components of the cellular chromatin. One question that remains is whether tethering of the KSHV genome is random or prone to localize on specific regions of the host chromosome. Infected cells harbor approximately 30–80 copies of the KSHV genome and ChIP-seq analysis has revealed at least 256 LANA-binding sites on the cellular genome, suggesting that not all LANA proteins may be involved in tethering the viral genome [26]. An interesting hypothesis is that the viral genome could be tethered to heterochromatin-enriched nuclear regions during latency and may relocate to a transcriptionally favorable nuclear compartment upon reactivation. Microscope analysis of the nuclear distribution of LANA in latently infected B cells showed that LANA preferentially associates with the border of heterochromatin, inviting speculation that close proximity to heterochromatin-rich regions of the host cell may be critical for the formation and maintenance of heterochromatin on the KSHV genome [98].

The structural organization of chromatin domains in the cellular genome plays an important role in the regulation cellular gene expression. Similarly, the chromatinized KSHV episome is also compartmentalized into different chromatin domains, which can interact with each other by a looping mechanism mediated by cellular factors that bind to the KSHV genome [99]. The maintenance of viral latency requires the separation of the latent genes from the lytic genes-encoding part of the KSHV genome so that the latent genes can be continuously expressed, while the lytic genes are repressed during latency. Lieberman’s group found that CTCF and the cohesion complex, which are known chromatin boundary factors separating active and inactive chromatin domains, bound to several sites of the KSHV genome and they are involved in the transcription regulation of both latent and IE genes during latency [100,101]. One of the CTCF-binding sites (CBS) can be found in the intron of the latent gene, LANA. Deletion of CBS disrupted cohesin binding to the KSHV genome and caused viral episome instability and increased expression of lytic genes [100]. In addition, chromatin conformation capture (3C) assays provided evidence for the first time that the latent LANA promoter physically interacts with the lytic RTA promoter during latency, which is mediated by the CTCF/cohesin complex in the intron of LANA [99]. In agreement with this, CBS mutation, siRNA depletion of CTCF or the cohesin complex component RAD21 diminished the interaction between these latent and lytic promoters and deregulated the latent gene expression program. This viral chromosome looping was also disrupted during lytic reactivation suggesting that the CTCF/cohesin-mediated looping in the viral genome is dynamic and involved in the regulation of latent and lytic gene expression [99]. The CTCF/cohesin complex in the intron of LANA also plays a critical role in the regulation of transcription elongation and nucleosome organization in the latency locus [102,103].

Every KSHV infected cell carries multiple copies of KSHV genomes, which raises the question whether they all have the same nucleosome structure during latency, which change simultaneously during reactivation or they show diversity. To answer this question a recent study used single-molecule footprinting assays called MAPit (Methyltransferase Accessibility Protocol for individual templates), which allows the detection of multiple chromatin states at selected loci within a cell population [104]. The chromatin architecture of the promoter of the constitutively expressed latent gene, LANA, the promoter of the IE gene, RTA and the promoter of the early gene, K2 was investigated. Their analysis showed diverse chromatin at each of these promoters, which ranged from closed to open conformations. Interestingly, the induction of lytic gene expression program resulted in the remodeling of the viral chromatin only on a fraction of the viral episomes. These results indicate that KSHV genomes possess diverse chromatin conformations in infected cells during both latency and reactivation. Furthermore, local chromatin condensations caused by epigenetic drift can restrict the expression of any viral genes irrespectively of what gene expression classes (IE, E, L or latent) they belong to [104].

## 4. Reprogramming of the Host Transcriptome during KSHV Infection

*In vitro* experiments have shown that KSHV infection of endothelial cells results in transcriptional reprogramming such that infected lymphatic endothelial cells are driven toward a blood vessel endothelial cell phenotype and vice versa [105,106]. In addition, KSHV infection can also induce the transcriptional reprogramming of lymphatic endothelial cells to mesenchymal cells [107]. These observations imply that viral factors may be involved by either deregulating the expression of cell type-specific transcription factors or modulating their functions, which results in altered cellular gene expression profile. Both LANA and viral miRNAs, for example, have been implicated to play a role in changing the expression of specific cellular genes encoding master transcription factors [106,108]. These viral factors can use different ways to regulate cellular genes. For example, LANA recruits DNA methyltransferases to specific cellular promoters such as that of H-cadherin and TGF-β type II receptor, resulting in hypermethylation and transcriptional repression of these promoters [32,109]. Because LANA has been shown to interact with a wide variety of chromatin-associated regulatory proteins, it can affect large number of cellular genes. Recent ChIP-seq analysis revealed 256 LANA-binding sites on the cellular genome, several of which are linked to p53-, TNF- or IFN-γ regulated genes [26].

KSHV encodes 12 miRNA genes that produce 25 mature miRNAs. These are small non-coding gene regulatory RNAs that can bind to mRNAs resulting in translational block or their degradation to repress genes [110]. A recent study showed that the KSHV miRNA miR-K12-11 shares significant sequence homology with the cellular miRNA called miR-155, which is often overexpressed in human tumors [111]. Strikingly, when miR-K12-11 was expressed in bone marrow cells, it caused the downregulation of Jarid2, a component of the PRC2 complex [111]. Thus, this viral miRNA can potentially deregulate the expression of PRC2 target genes such as that are involved in cell cycle control, for example, which can contribute to the development of KSHV-associated malignancies. Another KSHV miRNA, miR-K12-4-5p targets the retinoblastoma-like protein 2 (Rbl2), which is a repressor of the *de novo* DNA methyltransefarses DNMT3a and DNMT3b genes [112]. Expression of miR-K12-4-5p reduces Rbl2 expression resulting in the increased expression of DNMTs that can globally affect cellular gene expression in infected cells. PAN RNA of KSHV that is abundantly expressed in the nucleus during reactivation has been reported to downregulate the expression of many immunomodulatory genes [113]. PAN RNA can bind to gene promoters and recruit different histone modifying enzymes, which can be involved in the PAN RNA-mediated cellular gene regulation observed in infected cells [73,113]. 

Viral proteins often targets cellular histone modifying enzymes to relocate them on the cellular genome, modulate their enzymatic activity or their expression, which can affect the expression of a large number of cellular genes, all of which serve the needs of the virus for efficient infection of the host. Several KSHV proteins such as LANA, K8 and vIRF1 can bind to the histone acetyltransferase CBP/p300 and inhibit CBP/p300-mediated cellular transcription [37,114,115]. In contrast, RTA can use CBP to robustly activate gene transcription but it is still unclear how widely RTA uses CBP in activation of cellular genes [24]. K8 also interacts with the H3K9me3 histone demethylase JMJD2A and blocks its activity, which could play a role in the observed K8-mediated global cellular gene repression [77]. LANA forms a complex with the H3K9me1/2 histone demethylase JMJD1A in infected cells, which raises the question whether LANA uses JMJD1A to activate any of its cellular target genes [80].

EZH2, the H3K27me3 histone methyltransferase of the PRC2 complex has been shown to be overexpressed in KSHV-infected cells in Kaposi’s sarcoma (KS), suggesting that KSHV could be involved in EZH2 upregulation [116]. Indeed, KSHV infection of endothelial cells *in vitro* can induce the expression of EZH2, and this is mainly mediated by the KSHV latent proteins LANA and vFLIP via the upregulation of the NF-κB pathway [116]. Importantly, the increased expression of EZH2 turned out to be essential for KSHV-induced angiogenesis in KSHV-infected cells [116].

KSHV infection of B cells in human can lead to the development of the KSHV-associated primary effusion lymphoma (PEL), which is characterized by the disruption of the B-cell specific transcriptional program. Protein expression of several transcription factors that are essential for B-cell development is significantly altered in PEL cells compared to uninfected B-cells [117]. While PEL cells constitutively express IRF4, the protein level of other B-cell transcription factors such as Pax5, Oct-2, PU.1 and IRF‑8 was completely abolished [117]. The viral factors involved in the reprogramming of the B‑cell‑specific transcription factor network have not yet been identified.

## 5. Outlook

ChIP-on-chip analysis of the KSHV episome revealed distinct chromatin domains on the viral genome, which are characterized by different histone modification and DNA methylation patterns indicative of targeted recruitment of specific cellular chromatin modifying enzyme complexes (Figure 3). The benefit of such compartmentalization of the KSHV genome might be the use of common transcription regulatory mechanisms for expression of genes that have related functions during the lifecycle of the virus. Strikingly, the IE and most of E genes are marked by activating histone marks while the majority of late genes, which are transcribed only following viral DNA replication, are associated with heterochromatin that remains unaltered during the early phase of reactivation [50,51]. These observations suggest that the type of chromatin structure associated with each viral gene can play a role in the regulation of their expression. Two recent papers have reported that H2AX, an isoform of the canonical histone H2A and the phosphorylation of histone H3 at serine 10 residue are also components of the viral chromatin landscape and regulate the persistence of viral episome in infected cells as well as the reactivation of the lytic gene expression program, respectively [118,119]. These studies also demonstrate that we only see the tip of the iceberg regarding to the components and the organization of the KSHV chromatin in infected cells.

Currently, at least 130 different posttranslational modification sites have been identified on histones and there are at least 150 histone-modifying enzymes known [120]. Because of the unique DNA sequence elements, the close proximity of genes and overlapping gene regulatory regions in the KSHV genome, KSHV may utilize unique mechanisms to regulate its genome. Also, the modulation of the function of cellular chromatin modifying enzymes by viral proteins allows the reprogramming of the cellular transcriptome in a way that becomes beneficial for persistent infection as well as the development of KSHV-associated malignancies. It has been demonstrated that drugs targeting of histone modifying enzymes is a viable strategy for controlling HSV-1 and Human cytomegalovirus (HCMV) infections [121,122,123]. Therefore, identifying the relevant cellular and viral factors involved in the chromatin control of KSHV infection may bring new opportunities for pharmacological control of KSHV infection and KSHV-associated diseases.
